# Risk Factors for Posttraumatic Stress Disorder in Mexican Psychiatric Trauma Survivors

**DOI:** 10.62641/aep.v54i4.2253

**Published:** 2026-08-15

**Authors:** Laura Estefanía Mora Ortiz, Ilyamín Merlín García, Rebeca Robles García

**Affiliations:** ^1^Servicio de Psiquiatría, Hospital Alfredo Noboa Montenegro, 020101 Guaranda, Ecuador; ^2^Clinical Services, Ramón de la Fuente Muñiz National Institute of Psychiatry, 14370 Mexico City, Mexico; ^3^Center for Global Mental Health Research, Ramón de la Fuente Muñiz National Institute of Psychiatry–UNAM, 14370 Mexico City, Mexico

**Keywords:** PTSD, survivors, risk factor, sexual violence, women, mental disorders

## Abstract

**Background:**

There is extensive evidence linking specific sociodemographic and clinical factors to increased vulnerability for Posttraumatic Stress Disorder (PTSD). However, most studies have focused on community samples, and little is known about how such factors shape PTSD risk in clinical psychiatric settings in Latin America, where trauma exposure is highly prevalent. The present study addresses these gaps by examining sociodemographic characteristics and specific types of traumatic events linked to PTSD diagnosis among individuals receiving care at a tertiary mental health facility in Mexico City, Mexico.

**Methods:**

A cross-sectional, observational design was employed, enrolling 160 psychiatric patients. Participants were assessed for exposure to potentially traumatic events using the Life Events Checklist for DSM-5 (LEC-5). Probable PTSD was initially identified with the PTSD Checklist for DSM-5 (PCL-5), followed by diagnostic confirmation using the Clinician-Administered PTSD Scale for DSM-5 (CAPS-5). Sociodemographic data, including age, sex, and educational attainment, were also collected. Multivariate logistic regression was used to identify PTSD predictors, with a significance set at *p* < 0.05.

**Results:**

The frequency of PTSD in the sample was 31.9%. Multivariate logistic regression analysis revealed that sexual aggression was the strongest independent predictor of PTSD (odds ratio (OR) = 38.49; 95% confidence interval (CI): 10.59–139.95; *p* < 0.001). Other significant predictors included being female (OR = 8.09; 95% CI: 1.60–40.86; *p* < 0.001) and over 30 years old (OR = 2.86; 95% CI: 1.13–7.24; *p* < 0.05). The model demonstrated good overall fit (Pearson χ^2^ *p* = 0.673) and explained a moderate proportion of the variance (Nagelkerke R^2^ = 0.550).

**Conclusions:**

Sexual aggression, female sex, and older age were significantly associated with a PTSD diagnosis among psychiatric trauma survivors. These findings underscore the importance of incorporating trauma-informed care principles and systematic screening for sexual violence into mental health settings to improve diagnostic accuracy and optimize interventions for this population.

## Introduction

The term “Posttraumatic Stress” was coined in 1980 to refer to the acute emotional difficulty arising in response to an event experienced by an individual that disrupts their affective and cognitive state. Triggering events are usually catastrophic in nature, including accidents, the diagnosis of serious illnesses, or assaults intentionally caused by others (such as rape, terrorism, or domestic abuse). According to the most recent World Health Organization’s classification of mental disorders [1], Posttraumatic Stress Disorder (PTSD) is a mental health problem that can develop after exposure to an extremely threatening or horrific event or series of events. It is characterized by three clusters of symptoms. The first is persistent re-experiencing of the traumatic event through intrusive memories, flashbacks, or nightmares, often accompanied by intense emotional and physiological responses. The second is active avoidance of thoughts, memories, activities, situations, or people associated with the trauma. The third is a constant, heightened perception of current threat, manifested as hypervigilance or disproportionate startle responses. Symptoms persist for several weeks at least and cause significant impairment in personal, familial, social, educational, occupational, and other key areas of functioning.

Internationally, it is estimated that approximately 70% of adults will experience at least one traumatic event in their lifetime [2]; 20–45% of those exposed to significant traumas and 5–10% of those exposed to lesser traumas will develop PTSD [3]. The likelihood of developing the disorder varies depending on different factors, including:

(1) The type of traumatic event, with sexual trauma as a particularly high-risk factor [4, 5, 6];

(2) By sex, with substantial differences between women and men in terms of prevalence and symptomatology. Women have twice as high lifetime prevalence of PTSD compared to men despite a similar likelihood of exposure to traumatic events in the two sexes [7], which suggest the involvement of biological (hormonal) and gender-related psychosocial differences [8]; and

(3) The presence of other mental disorders. Psychiatric inpatients and outpatients significantly exceed lifetime prevalence of PTSD in general population [9, 10, 11, 12]. The lifetime prevalence of PTSD is around 30% among psychiatric inpatients; from those 60% had one or two other psychiatric diagnoses and 26% had more than three other diagnoses; the most frequent current comorbid psychiatric disorder as a function of PTSD status was major depression (51.3%) [12].

The coexistence of PTSD with other psychiatric comorbidities has also been associated with an increased risk of suicidal behavior [13] morbidity and premature mortality [14], underscoring the clinical severity of this condition. However, PTSD is frequently underdiagnosed or inadequately identified and treated even in clinical contexts. The identification of risk and protective factors could provide crucial information for understanding the development of PTSD in psychiatric populations and the need for targeted evaluation and treatment measures.

### PTSD in Psychiatric Populations

Sociodemographic characteristics, such as being female, low socioeconomic status, and lower educational attainment, increase vulnerability to PTSD in individuals with a history of mental disorders such as depression or anxiety [15]. Additionally, psychiatric hospitalization itself can represent a significant risk factor for the development of PTSD, especially in patients with affective disorders and those exposed to violence during hospitalization [16].

Despite extensive evidence linking sociodemographic and clinical factors to increased vulnerability for PTSD, most studies have focused on community samples and have relied primarily on self-report instruments rather than clinician-administered diagnostic tools. Moreover, little is known about how sociodemographic characteristics and specific traumatic events shape PTSD risk in clinical psychiatric settings in Latin America, where trauma exposure is highly prevalent. The present study addresses these gaps by examining sociodemographic predictors and trauma profiles associated with clinician-confirmed PTSD in a Mexican psychiatric population, providing region-specific evidence for trauma-informed assessment and clinical decision-making.

## Materials and Methods

This is an observational, prospective, cross-sectional, comparative, and analytical study, with consent psychiatric outpatients and inpatients adults aged 18 receiving mental health care at the National Institute of Psychiatry, a tertiary level psychiatric facility in Mexico City, Mexico. From them, we excluded those who did not report at least one traumatic experience (according to Life Events Checklist for DSM-5 (LEC-5), see description below), and eliminated those who did not complete the rest of the evaluations. Therefore, all participants were considered psychiatric trauma survivors that might or not develop PTSD. Thus, frequency of PTSD was expected to be higher than in general psychiatric population (which per se is more likely to have PTSD than general population). The specific period of sample collection was 1 March 2023 to 30 December 2023.

### Measures

Sociodemographic variables included age, sex, marital status, years of schooling completed, and occupational status. Exposure to traumatic events was assessed using the LEC-5 [17]. LEC-5 is a self-reported scale to indicate whether a participant has experienced or not (yes/no) one or more potentially traumatic events. It includes sixteen options of experience and one additional item for other stressful events not captured by the previous items.

PTSD Checklist for DSM-5 (PCL-5) [18] was used as a screening measure for PTSD. PCL-5 is a 20-item self-report instrument that assesses DSM-5 symptoms of PTSD, which can be scored in different ways. In the present study, the total score was calculated (range = 0–80) by summing the scores of all the items. In Mexican mental health service users, the empirically derived optimal cutoff score for probable PTSD is 27 [19].

The diagnostic confirmation of PTSD was made using the Clinician-Administered PTSD Scale for DSM-5 (CAPS-5) [20] among those who had scores ≥27 in PCL-5. CAPS-5 is a structured clinical interview that includes questions about the respondent’s most traumatic event reported in LEC-5 (index traumatic event) in order to assess severity of such stressor and its subjective experience, allowing the diagnosis of PTSD to be established according to the criteria of the DSM-5. It is a 30-item structured interview, considered the gold standard for current and lifetime diagnosis of PTSD.

### Procedures

Psychiatrics and psychiatry residents were informed about the project and asked to refer out- and inpatient potential participants interested in being part of the study. Following contact with potential participants, a verbal invitation was extended, explaining the objectives, implications, risks, benefits, voluntariness, and confidentiality of the study. Those who accepted and signed the corresponding consent form were included.

An initial assessment was performed to obtain sociodemographic and clinical data. LEC-5 was administered to evaluate the presence of potential traumatic events. Patients with no traumatic events were excluded from the study. Then, PCL-5 was administered to the study sample (with at least one potential traumatic event). CAPS-5 was administered to those who exceeded PCL-5 threshold to confirm PTSD and integrate two study groups: patients with and without PTSD.

All assessments conducted by a resident of the specialty in psychiatry with previous specific training to apply the CAPS-5 (“CAPS-5 Training Curriculum” of the US: Department of Veterans Affairs, National Center for PTSD: https://www.ptsd.va.gov/professional/continuing_ed/caps5_clinician_training.asp).

### Data Analysis

Descriptive information included frequencies and percentages. All variables were compared between patients with and without PTSD using Chi-square tests (χ^2^). After comparative analyses, a logistic regression was performed using the variables that were different between groups as possible predictors of the presence of PTSD. The Pearson chi-square test was used to determine the model’s goodness of fit, and the Nagelkerke R^2^ was determined to identify the variance explained by the model. Regression coefficients (β), standard deviations of β, odds ratios (OR), and 95% confidence intervals (CIs) are reported. All analyses were performed using SPSS version 21 (IBM Corporation, Armonk, NY, USA) for Windows, PC, and the alpha value for tests was set at *p* < 0.05.

## Results

Total sample for the study consisted of 160 patients who had survived one or more traumatic events. Fifty-one of them (31.9%) met the diagnostic criteria for PTSD. Table 1 presents the demographic and clinical characteristics of the total sample and each group of trauma survivors (with and without PTSD). As can be seen, statistically significant differences were observed in the distribution by sex (χ^2^(1) = 14.20, *p* < 0.001), with more women than men in the PTSD group. Moreover, the mean age was significantly higher in the PTSD group, with more than half of the patients being approximately thirty years old. No significant differences were identified between the groups as regards marital status (χ^2^), educational attainment, occupation, or number of previous psychiatric hospitalizations.

**Table 1.** **Sample description: Sociodemographic and clinical characteristics of trauma survivors with and without PTSD (n = 160)**.

Variable		Total n = 160	With PTSD n = 51 (31.9)	Without PTSD n = 109 (68.1)	Comparison between groups
Sex, n (%)					χ^2^(1) = 14.20, *p * < 0.001
	Female	125 (78.1)	49 (96.1)	76 (69.7)	
	Male	35 (21.9)	2 (3.9)	33 (30.3)	
Age, n (%)					χ^2^(1) = 4.22, *p* = 0.040
	≤30 years old	94 (58.8)	24 (47.1)	70 (64.2)	
	>30 years old	66 (41.2)	27 (52.9)	39 (37.8)	
Marital status, n (%)					χ^2^(3) = 3.153, *p* = 0.369
	Single	117 (73.1)	34 (66.7)	83 (76.1)	
	Married	21 (13.1)	7 (13.7)	14 (12.8)	
	Common-law union	11 (6.9)	4 (7.8)	7 (6.4)	
	Separation/divorce	11 (6.9)	6 (11.8)	5 (4.6)	
Education, n (%)					χ^2^(5) = 6.240, *p* = 0.284
	Elementary	6 (3.8)	3 (5.9)	3 (2.8)	
	Junior high school	18 (11.2)	3 (5.9)	15 (13.8)	
	Senior high school	74 (46.2)	25 (49.0)	49 (44.9)	
	Technical course	10 (6.2)	1 (1.9)	9 (8.2)	
	Bachelor’s degree	46 (28.8)	16 (31.4)	30 (27.5)	
	Postgraduate	6 (3.8)	3 (5.9)	3 (2.8)	
Occupation, n (%)					χ^2^(3) = 3.191, *p* = 0.671
	Student	51 (31.9)	13 (25.4)	38 (34.9)	
	Employee	46 (28.7)	16 (31.4)	30 (27.5)	
	Unemployed	34 (21.3)	11 (21.6)	23 (21.1)	
	Homemaker	28 (17.5)	11 (21.6)	17 (15.6)	
	Retiree/pensioner	1 (0.6)	0 (0)	1 (0.9)	
Hospitalizations, n (%)					χ^2^(3) = 3.916, *p* = 0.271
	None	123 (76.9)	44 (86.3)	79 (72.5)	
	One	30 (18.7)	6 (11.8)	24 (22.0)	
	Two	6 (3.8)	1 (1.9)	5 (4.6)	
	Three	1 (0.6)	0 (0)	1 (0.9)	

Note: No comparison was made between groups with fewer than five subjects per group Traumatic Events in Psychiatric Patients with and without PTSD. PTSD, Posttraumatic Stress Disorder.

Table 2 describes the frequency of traumatic events in the total sample and by the study group (with and without PTSD). Regarding the traumatic events experienced, sexual assault was reported more frequently by the PTSD group than by the group without PTSD. In contrast, other traumatic events, including physical assault, natural disasters, accidents, robberies, and violent death of a third party, did not show significant differences between groups.

**Table 2.** **Traumatic events in trauma survivors with and without PTSD**.

Variable	Total sample n = 160	With PTSD n = 51	Without PTSD n = 109	Comparison with/without PTSD
Sexual Agression, n (%)	81 (50.6)	48 (94.1)	33 (30.3)	χ^2^(1) = 56.653, *p * < 0.001
Physical Aggression, n (%)	70 (43.8)	24 (47.1)	46 (42.2)	NS
Natural Disaster, n (%)	28 (17.5)	13 (25.5)	15 (13.8)	NS
Transport Accident, n (%)	26 (16.3)	11 (21.6)	15 (13.8)	NS
Armed Assault, n (%)	19 (11.9)	9 (11.6)	10 (9.2)	NS
Serious Injury/Death of Another Person, n (%)	9 (5.6)	5 (9.8)	4 (3.7)	–

Notes: Columns do not sum 100% give each participant might have more than one traumatic experience. No comparison was made between groups with fewer than five subjects per group. NS, non-significant contrast; PTSD, Posttraumatic Stress Disorder.

In the logistic regression model (Table 3). As can be seen, sexual assault was the most significant predictor of PTSD diagnosis, with an OR = 38.49 (95% CI: 10.59–139.95; *p* < 0.001). Likewise, being female was associated with a more than eightfold increase in the likelihood of developing PTSD (OR = 8.09; 95% CI: 1.60–40.86; *p* < 0.001). Finally, being over 30 years old was also significantly associated with a higher risk of PTSD (OR = 2.86; 95% CI: 1.13–7.24; *p* < 0.05). The model showed a good overall fit (Pearson’s χ^2^*p* = 0.673) and moderate explanatory power (Nagelkerke’s R^2^ = 0.550).

**Table 3.** **Logistic Regression Model for PTSD in psychiatric trauma survivors (n = 160)**.

Variable	β	OR	95% CI
Sex – women	2.09***	8.09	1.60–40.86
Age – >30 years old	1.05*	2.86	1.13–7.24
Sexual aggression – yes	3.65***	38.49	10.59–139.95

*p < 0.05; ***p < 0.001. Pearson Chi square *p* = 0.673; Nagelkerke R^2^ = 0.550. OR, odds ratio; CI, confidence interval; PTSD, Posttraumatic Stress Disorder.

## Discussion

This study examined sociodemographic characteristics, risk factors, and types of traumatic events among trauma survivor psychiatric patients with and without a current diagnosis of PTSD. First, a frequency of PTSD of 31.9% was observed in this particular clinical sample, which is consistent with previous evidence of high rates of PTSD in specialized psychiatric settings [12]. This comorbidity, in addition to complicating diagnosis, might negatively impact therapeutic response, prognosis, and overall functioning. PTSD can be conceptualized both as a primary disorder, a direct result of the traumatic experience, and as a secondary manifestation that emerges in individuals with a prior vulnerability [21].

Given PTSD might be an underdiagnosed mental health condition in tertiary psychiatric care services due to symptomatic overlaps with other disorders such as mood or anxiety disorders [22], this result highlights the importance of implementing assessment protocols that systematically include the exploration of traumatic history among psychiatric patients. Omitting this information can lead to nonspecific or ineffective therapeutic interventions. Informed assessment of trauma should therefore be a diagnostic and therapeutic priority, especially in populations with risk factors associated with PTSD cooccurrence.

According to Kilpatrick *et al*. [23] sexual assaults, especially those perpetrated in close interpersonal contexts, might produce greater psychological impact than other types of traumatic events, such as natural disasters or accidents. In our study, sexual assault emerged as the most robust risk factor for PTSD diagnosis, showing that psychiatric patients with a history of sexual assault might be thirty-eight times more likely to develop the disorder. The magnitude of sexual violence among psychiatric patients, notably higher than that reported in general population [24, 25], highlights its clinical and psychopathological relevance in mental health service users.

Moreover, the experience of sexual assaults is more frequent among women, and being female was another risk factor for PTSD in our sample of psychiatric trauma survivors, increasing eight times the likelihood of having this mental disorder. This is in line with the widely documented women’s higher risk for PTSD in general population [26, 27] and raises that being female might constitute a marker of social and clinical risk deeply intertwined with contextual factors. It is estimated that between 15% and 25% of women will experience at least one episode of sexual assault in their lifetime, underlining the need to implement comprehensive prevention, detection, and treatment strategies within mental health systems [28] and reinforcing gender-sensitive approaches in diagnostic and intervention processes [29].

Another relevant risk factor for PTSD in our sample was age, with participants over thirty years old having an almost three times higher diagnostic probability. This finding aligns with epidemiological evidence on the course of PTSD throughout the life cycle. In the National Epidemiologic Survey on Alcohol and Related Conditions (NESARC-II), a significantly higher prevalence of PTSD was observed in young (4.3%), and middle-aged (5.2%) adults compared to older adults (≥65 years, 2.6%) [30]. These results suggest that the risk of developing PTSD might be determined by both cumulative exposure to trauma and reduced adaptive flexibility. In this respect, age should be considered as a relevant clinical factor in diagnostic evaluation and in the formulation of interventions, which implies adapting therapeutic approaches to the evolutionary characteristics and specific needs of each age group.

The regression model showed a moderately high explanatory power, indicating that the three variables considered—sex, age, and sexual aggression—explain a substantial proportion of the variance in the diagnosis of PTSD. However, the model does not fully explain the phenomenon. This suggests the involvement of other factors not included in this study. In this sense, the possible analytical approach employed in the study is relatively basic, and key potential confounders (e.g., psychiatric comorbidities, dissociation and trauma severity, cumulative trauma exposure) and psychosocial factors (e.g., coping, resilience, social support) are not fully considered, which in turn may inflate the observed associations (e.g., extremely high ratio for sexual aggression).

Moreover, although the findings of the present study provide clinically relevant evidence, they should be interpreted with caution. First, cross-sectional design prevents the establishment of causal relationships and limits interpretability. Second, the sample size and the fact that it was drawn from a single psychiatric center limit the generalizability of results. Finally, the use of self-administered scales to assess traumatic events may have introduced memory or avoidance biases, particularly in patients with dissociative symptoms.

Future studies should employ larger samples and longitudinal designs allowing the evaluation of the influence of moderating and mediating biopsychosocial variables, as well as clinical trajectories of recovery or chronicity in psychiatric trauma survivors.

## Conclusions

Sexual aggression, female sex, and older age were significantly associated with a PTSD diagnosis among Mexican psychiatric trauma survivors. These findings underscore the importance of incorporating trauma-informed care principles and systematic screening for sexual violence and PTSD into mental health settings to improve diagnostic accuracy and optimize interventions for this population, particularly in women. Understanding the clinical and sociodemographic profiles associated with this disorder is fundamental to designing more specific, effective, and culturally relevant interventions.

## Availability of Data and Materials

De-identified data included in this study can be available by contacting the corresponding author upon reasonable request.

## References

[1] World Health Organization (2022). *International Classification of Diseases Eleventh Revision (ICD-11)*.

[2] Benjet C, Bromet E, Karam EG, Kessler RC, McLaughlin KA, Ruscio AM (2016). The epidemiology of traumatic event exposure worldwide: results from the World Mental Health Survey Consortium. *Psychological medicine*.

[3] Andersen HS (1998). The epidemiology of post-traumatic stress disorders. *Ugeskrift for laeger*.

[4] Atwoli L, Stein DJ, Koenen KC, McLaughlin KA (2015). Epidemiology of posttraumatic stress disorder: Prevalence, correlates, and consequences. *Current opinion in psychiatry*.

[5] Jonas S, Bebbington P, McManus S, Meltzer H, Jenkins R, Kuipers E (2011). Sexual abuse and psychiatric disorder in England: Results from the 2007 Adult Psychiatric Morbidity Survey. *Psychological medicine*.

[6] García-Esteve L, Torres-Giménez A, Canto M, Roca-Lecumberri A, Roda E, Velasco ER (2021). Prevalence and risk factors for acute stress disorder in female victims of sexual assault. *Psychiatry research*.

[7] Perrin M, Vandeleur CL, Castelao E, Rothen S, Glaus J, Vollenweider P (2014). Determinants of the development of post-traumatic stress disorder, in the general population. *Social psychiatry and psychiatric epidemiology*.

[8] Robles-García R, Fresán A, Yoldi M (2020). Posttraumatic stress disorder in urban women. *Current opinion in psychiatry*.

[9] Kessler RC, Sonnega A, Bromet E, Hughes M (1995). Posttraumatic stress disorder in the National Comorbidity Survey. *Archives of general psychiatry*.

[10] Koenen KC, Ratanatharathorn A, Ng L, McLaughlin KA, Bromet EJ, Stein DJ (2017). Posttraumatic stress disorder in the World Mental Health Surveys. *Psychological medicine*.

[11] Seong A, Cho SE, Na KS (2023). Prevalence and correlates of comorbid posttraumatic stress disorder in schizophrenia-spectrum disorder: A systematic review and meta-analysis. *Psychiatry investigation*.

[12] McFarlane AC, Bookless C, Air T (2001). Posttraumatic stress disorder in a general psychiatric inpatient population. *Journal of traumatic stress*.

[13] Akbar R, Arya V, Conroy E, Wilcox HC, Page A (2023). Posttraumatic stress disorder and risk of suicidal behavior: A systematic review and meta-analysis. *Suicide & life-threatening behavior*.

[14] Nilaweera D, Phyo AZZ, Teshale AB, Htun HL, Wrigglesworth J, Gurvich C (2023). Lifetime posttraumatic stress disorder as a predictor of mortality: A systematic review and meta-analysis. *BMC psychiatry*.

[15] Hyland P, Vallières F, Cloitre M, Ben-Ezra M, Karatzias T, Olff M (2021). Trauma, PTSD, and complex PTSD in the Republic of Ireland: Prevalence, service use, comorbidity, and risk factors. *Social psychiatry and psychiatric epidemiology*.

[16] Martinaki S, Kostaras P, Mihajlovic N, Papaioannou A, Asimopoulos C, Masdrakis V (2021). Psychiatric admission as a risk factor for posttraumatic stress disorder. *Psychiatry research*.

[17] Weathers FW, Blake DD, Schnurr PP, Kaloupek DG, Marx BP, Keane TM (2013). The Life Events Checklist for DSM-5 (LEC-5). https://www.ptsd.va.gov/professional/assessment/te-measures/life_events_checklist.asp.

[18] Weathers FW, Litz BT, Keane TM, Palmieri PA, Marx BP, Schnurr PP (2013). The PTSD Checklist for DSM-5 (PCL-5). https://www.ptsd.va.gov/professional/assessment/adult-sr/ptsd-checklist.asp.

[19] Martínez-Levy GA, Bermúdez-Gómez J, Merlín-García I, Flores-Torres RP, Nani A, Cruz-Fuentes CS (2021). After a disaster: Validation of PTSD Checklist for DSM-5 and the four- and eight-item abbreviated versions in mental health service users. *Psychiatry research*.

[20] Weathers FW, Blake DD, Schnurr PP, Kaloupek DG, Marx BP, Keane TM (2013). The Clinician-Administered PTSD Scale for DSM-5 (CAPS-5). https://www.ptsd.va.gov/professional/assessment/adult-int/caps.asp.

[21] van der Kolk B (2000). Posttraumatic stress disorder and the nature of trauma. *Dialogues in clinical neuroscience*.

[22] Escalona R, Tupler LA, Saur CD, Krishnan KR, Davidson JR (1997). Screening for Trauma History on an Impatient affective-disorder unit: a pilot study. *Journal of traumatic stress*.

[23] Kilpatrick DG, Resnick HS, Milanak ME, Miller MW, Keyes KM, Friedman MJ (2013). National estimates of exposure to traumatic events and PTSD prevalence using DSM-IV and DSM-5 criteria. *Journal of traumatic stress*.

[24] Khalifeh H, Moran P, Borschmann R, Dean K, Hart C, Hogg J (2015). Domestic and sexual violence against patients with severe mental illness. *Psychological medicine*.

[25] Kaul A, Connell-Jones L, Paphitis SA, Oram S (2024). Prevalence and risk of sexual violence victimization among mental health service users: A systematic review and meta-analyses. *Social psychiatry and psychiatric epidemiology*.

[26] Tolin DF, Foa EB (2006). Sex differences in trauma and posttraumatic stress disorder: A quantitative review of 25 years of research. *Psychological bulletin*.

[27] Olff M, Langeland W, Draijer N, Gersons BP (2007). Gender differences in posttraumatic stress disorder. *Psychological bulletin*.

[28] Yoo Y, Park HJ, Park S, Cho MJ, Cho SJ, Lee JY (2018). Interpersonal trauma moderates the relationship between personality factors and suicidality of individuals with posttraumatic stress disorder. *PloS one*.

[29] Guzmán-Parra J, Martínez-García AI, Guillén-Benítez C, Castro-Zamudio S, Jiménez-Hernández M, Moreno-Küstner B (2016). Factors related to psychiatric calls to the Prehospital Emergency care services in Málaga (Spain). *Salud Mental*.

[30] Reynolds K, Pietrzak RH, Mackenzie CS, Chou KL, Sareen J (2016). Post-traumatic stress disorder across the adult lifespan: Findings from a nationally representative survey. *The American journal of geriatric psychiatry : official journal of the American Association for Geriatric Psychiatry*.

